# Genome-Wide Estimates of Mutation Rates and Spectrum in *Schizosaccharomyces pombe* Indicate CpG Sites are Highly Mutagenic Despite the Absence of DNA Methylation

**DOI:** 10.1534/g3.115.022129

**Published:** 2015-11-10

**Authors:** Megan G. Behringer, David W. Hall

**Affiliations:** Department of Genetics, University of Georgia, Athens, Georgia 30602

**Keywords:** mutation accumulation, insertion bias, cytosine mutation

## Abstract

We accumulated mutations for 1952 generations in 79 initially identical, haploid lines of the fission yeast *Schizosaccharomyces pombe*, and then performed whole-genome sequencing to determine the mutation rates and spectrum. We captured 696 spontaneous mutations across the 79 mutation accumulation (MA) lines. We compared the mutation spectrum and rate to a recently published equivalent experiment on the same species, and to another model ascomycetous yeast, the budding yeast *Saccharomyces cerevisiae*. While the two species are approximately 600 million years diverged from each other, they share similar life histories, genome size and genomic G/C content. We found that *Sc. pombe* and *S. cerevisiae* have similar mutation rates, but *Sc. pombe* exhibits a stronger insertion bias. Intriguingly, we observed an increased mutation rate at cytosine nucleotides, specifically CpG nucleotides, which is also seen in *S. cerevisiae*. However, the absence of methylation in *Sc. pombe* and the pattern of mutation at these sites, primarily C → A as opposed to C → T, strongly suggest that the increased mutation rate is not caused by deamination of methylated cytosines. This result implies that the high mutability of CpG dinucleotides in other species may be caused in part by a methylation-independent mechanism. Many of our findings mirror those seen in the recent study, despite the use of different passaging conditions, indicating that MA is a reliable method for estimating mutation rates and spectra.

Spontaneous mutation is the fuel for evolution and the ultimate source of all genetic differences within and between species. A complete understanding of the mutational process, both in terms of the rate at which different mutations arise, including kind and location, and the determination of their fitness effects, is thus critical to understanding genetic variation at all levels. However, elucidating the important parameters of mutation is difficult for two reasons. First, spontaneous mutations occur infrequently, making it difficult to obtain large samples of spontaneous mutations to robustly detect patterns. One workaround is to artificially increase the mutation rate using chemical mutagens and X-rays (Muller 1930; Greene *et al.* 2003; Blumenstiel *et al.* 2009), or genetic methods such as repair pathway knock-outs (Hoffman *et al.* 2004; Denver *et al.* 2006). However, it is clear that such manipulations bias the mutational spectrum in various ways (Koornneeff *et al.* 1982; Greene *et al.* 2003). Another way to deal with the rarity of spontaneous mutations is to examine genetic differences between individuals, populations or species that have arisen via mutation. Unfortunately, because many mutations are acted upon by natural selection, this raises the second main difficulty with studying spontaneous mutations: the observed genetic variation has been acted upon by selection and is thus a biased sample of spontaneous mutations (Nachman and Crowell 2000; Ho *et al.* 2005). Exacerbating this problem is the finding that sites in the genome previously thought to be essentially free of selection, such as those in intronic regions, intergenic regions and fourfold redundant codon positions are, in fact, often constrained by selection, making the study of mutation at these sites biased by selection (Andolfatto 2005; Hershberg and Petrov 2008).

One approach that has been employed to overcome the problems of rarity and selection in studying spontaneous mutations is the mutation accumulation (MA) experiment. MA experiments maintain multiple, initially identical lines at very low effective population sizes for many generations (Halligan and Keightley 2009). Lines accumulate spontaneous mutations at rates proportional to their occurrence since selection is ineffective at enriching for beneficial or decreasing deleterious mutations, unless their fitness effects are large (Sung *et al.* 2012a). Each line accumulates only a few mutations but, by having numerous lines, several hundred mutations can be captured across the lines. Whole-genome sequencing (WGS) of the MA lines allows mutations to be identified and used to estimate the frequency and spectrum of spontaneous mutation. This approach has been used to examine spontaneous mutations in MA lines in a number of eukaryotic species including *Arabidopsis thaliana*, *Caenorhabditis elegans*, *Chlamydomonas reinhardtii*, *Dictyostelium discoideum*, *Drosophila melanogaster*, *Paramecium tetraurelia*, *Saccharomyces cerevisiae* and *Schizosaccharomyces pombe* (Keightley *et al.* 2009; Denver *et al.* 2012; Ness *et al.* 2012; Rutter *et al.* 2012; Saxer *et al.* 2012; Sung *et al.* 2012b; Zhu *et al.* 2014; Farlow *et al.* 2015).

The haploid, fission yeast *S. pombe* was originally isolated from millet beer, and is an important model organism in molecular biology (Leupold 1950; Humphrey 2000; Wood *et al.* 2002). While both are ascomycete yeasts, *Sc. pombe* is a distant relative of *S. cerevisiae*, with a divergence time of 600–1200 million years (Heckman *et al.* 2001; Douzery *et al.* 2004). Even so, they exhibit similar life histories as single-celled, sexual yeasts, and are cultured in the lab using essentially identical methods. In addition, they have similar genomic G/C content (36.06% in *Sc. pombe* *vs.* 38.29% in *S. cerevisiae*) and genome size [13.8 Mb in *Sc. pombe* (Wood *et al.* 2002) *vs.* 12.1 Mb in *S. cerevisiae* (Goffeau *et al.* 1996)]. While similar, they differ in one feature that is expected to have implications for their mutation rate and spectrum: *Sc. pombe* is a haploid as opposed to a diploid species in nature. The similarities and difference between these two yeast species allowed us to make several *a priori* predictions concerning mutation rate and spectrum in *Sc. pombe*. Before listing these predictions, a brief note on our terminology is useful: G/C and A/T indicate G or C and A or T, and are used when discussing nucleotide composition of regions of the genome; adjacent nucleotides in a single strand of DNA are indicated by the juxtaposition of the base designations with the 5′ nucleotide on the left, *e.g.*, CGA indicates a C nucleotide adjacent to a G nucleotide adjacent to an A nucleotide, with the A in the 3′ position; dinucleotides in the same strand that are Watson-Crick partners when in different strands, are indicated as CpG, GpC, ApT, and TpA to clarify that the nucleotides are in same strand; and base pairs in a DNA double helix are indicated with a colon, *e.g.*, C:G.

Our first prediction was that the genome-wide single-nucleotide mutation (SNM) rate would be higher in *Sc. pombe* than in *S. cerevisiae* based on estimates from reporter genes that average 8.2 × 10^−10^ single nucleotide substitutions per base per generation (summarized in Lynch 2010), which is 4.8-fold higher than the most recent estimate in *S. cerevisiae* (Zhu *et al.* 2014).

Our second prediction was that most of the SNM biases, which are the relative rates of mutations among the different base pairs, in *Sc. pombe* would generally be the same as in *S. cerevisiae*. This prediction comes from the fact that the genomic G/C content of *Sc. pombe* (36.06%) is similar to that of *S. cerevisiae* (38.29%). Interestingly, the SNM biases observed in *S. cerevisiae* predict a lower G/C content (32%) than observed (Zhu *et al.* 2014), suggesting another force acts on G/C content. One possibility is selection. If weak selection drives G/C content to a higher equilibrium than predicted based on SNM bias in both species, then we expect *Sc. pombe* to be closer to the mutation-bias equilibrium than *S. cerevisiae*, *i.e.*, lower, as observed, because of its smaller effective population size (calculated using previous estimates of diversity and mutation rate), which reduces the efficacy of selection. The SNM bias in *S. cerevisiae* shows an elevated mutation rate of C:G base pairs (Zhu *et al.* 2014). A major surprise concerning the elevated mutation rate at C:G base pairs was the finding that their mutagenicity is affected by trinucleotide context. Specifically, when C:G is the middle base pair in CCG (equivalent to CGG) and TCG (equivalent to CGA) trinucleotides, the mutation rate is elevated even more than for other C:G base pairs. Both of these trinucleotides include a CpG dinucleotide, which is a well-characterized target for methyl-transferases (Bestor and Verdine 1994). The bias at CpG dinucleotides in *S. cerevisiae* was in the C → T direction. For this reason, the finding of elevated C:G mutation at CCG and TCG trinucleotides was interpreted as indicating a very low occurrence of DNA methylation in *S. cerevisiae* (Zhu *et al.* 2014), in agreement with a previous study (Tang *et al.* 2012). *Sc. pombe* is believed to lack DNA methylation so we did not expect to see an elevation of the G:C mutation rate at CpG sites (Antequera *et al.* 1984).

Our third prediction was that among small insertions and deletions (indels) there would be an insertion bias. Small indels occur primarily at microsatellites, presumably primarily due to slippage of the DNA polymerase during replication (Levinson and Gutman 1987). In *S. cerevisiae*, indels of less than 50 bp were moderately biased toward deletions (18 deletions *vs.* eight insertions) across the diploid MA lines (Zhu *et al.* 2014). However, analysis of indels in haploid MA lines of *S. cerevisiae* showed a bias toward insertions (34 insertions and eight deletions at microsatellite loci) (Lynch *et al.* 2008). Attributing this insertion bias to haploidy is complicated by the fact that many of the haploid MA lines reverted to diploidy over the course of the MA experiment (Lynch *et al.* 2008). Nevertheless, *Sc. pombe* is a natural haploid and was passaged as such in our MA experiment, and so we hypothesized that we would see a bias toward insertions for small indels in this species.

Our fourth prediction was that mutations resulting from nonrecombinational repair of double-strand breaks would be more common in haploid *Sc. pombe* than in diploid *S. cerevisiae*. Nonrecombinational repair is substantially more mutagenic than recombinational repair (Daley *et al.* 2005). In a diploid cell, there is always a nonsister homolog present that can be used for recombinational repair. However, in a haploid cell, recombinational repair is only possible during the S or G2 phase of the cell cycle, when a sister chromatid is present. Repair of double-strand breaks is thus expected to be more error-prone in a haploid because of the higher likelihood of using nonrecombinational repair. While double-strand breaks cannot be directly observed or always inferred with certainty in the MA framework, their occurrence can be indicated, if they are inaccurately repaired, by the presence of multiple mutations in close proximity (Strathern *et al.* 1995; Holbeck and Strathern 1997). In diploid *S. cerevisiae*, three double mutations, which consist of two SNMs adjacent to one another, were observed across the MA lines (Zhu *et al.* 2014). The relative occurrence of double SNMs was thus ∼0.35% compared to SNMs. In addition, there were five “complex mutations”, in which multiple SNMs, and often small indels, were in close proximity, giving a relative occurrence of ∼0.58% compared to SNMs. If both double and complex mutations were due to error-prone double-strand break repair, then the observed number of these mutations accounted for ∼1% of SNMs. The haploid MA experiment in *S. cerevisiae* was too small to detect such rare events (Lynch *et al.* 2008). However, our MA experiment is large enough that we predicted we would see an increase in the rate of double mutations and complex mutations in *Sc. pombe* compared to *S. cerevisiae*, assuming these events are indeed caused by repair of double-stranded breaks. Our prediction was somewhat tempered by the fact that *Sc. pombe* spends only ∼10% of its cell cycle in the G1 phase when growing exponentially (Knutsen *et al.* 2011), and is thus haploid for a minority of the time, though this proportion can increase substantially when growth slows (Nasmyth 1979). An average, exponentially growing, *Sc. pombe* strain, is thus expected to have two copies of its genome in each cell 90% of the time, and a single copy 10% of the time, implying an average ploidy of 1.9 genomes. *S. cerevisiae*, in contrast, is in G1 (with two copies of each DNA region) about 25–35% of the time when growing exponentially (Slater *et al.* 1977; Brewer *et al.* 1984), implying an average ploidy of 3.4 genomes. These data imply that a double-strand break in *Sc. pombe* will be in a cell containing one homologous copy for possible recombinational repair about 90% of the time, and will have no template for repair about 10% of the time. In contrast, a double-strand break in *S. cerevisiae* will always have at least one copy, and will have three copies ∼70% of the time. So, while *Sc. pombe* may have a copy available for repair up to 90% of the time, the average number of copies available for repair is about 2.5 times less (0.9 *vs.* 2.4 extra copies) than in *S. cerevisiae*.

Our final *a priori* prediction was that we would not observe aneuploidy in *Sc. pombe*. Since *Sc. pombe* is haploid, loss of a single chromosome (nullisomy) results in loss of all copies of the genes on that chromosome, which would likely be lethal and thus unobservable. In addition, gain of a single chromosome (disomy) would result in a doubling of gene dose for all genes on the chromosome, which is a larger increase than the 1.5-fold increase that occurs with trisomy in diploid *S. cerevisiae*. If deleterious effects due to gene dosage were increased with larger differences in dosage across genes, this would further reduce the likelihood of observing aneuploidy. In addition, *Sc. pombe* has only three chromosomes, implying that many more genes would be affected by an aneuploidy event than in *S. cerevisiae*, which has a similar genome size but 16 chromosomes. The only instance of aneuploidy reported in previous work in *Sc. pombe* is disomy of chromosome III, which was highly deleterious (Niwa *et al.* 2006). For these reasons, we predicted we would see no nullisomy, and likely no disomy in any of our MA lines.

One month prior to submission of our results, Farlow and colleagues (Farlow *et al.* 2015) published the results of an MA experiment on *Sc. pombe* of essentially identical scope. While the inadvertent duplication of effort is unfortunate, their results allow us to investigate the repeatability of the MA framework for determining estimates of parameters of mutation. Their study utilized a different starting strain, different media, different growth temperatures, and longer time between transfers, allowing us to determine whether these factors together alter parameter estimates.

This study presents genome-wide estimates of mutational parameters for *Sc. pombe*. We examined 79 MA lines, cultivated as haploids for an average of 1952 generations. We were able to identify a total of 696 mutations. These mutations allowed us to calculate precise estimates of the mutation rate and spectrum for *Sc. pombe*, test our *a priori* predictions and determine the repeatability of MA for parameter estimation.

## Materials and Methods

### Mutation accumulation lines

*Sc. pombe* MA lines were passaged in the same manner as described for *S. cerevisiae* (Joseph and Hall 2004). Briefly, the haploid ancestral line, 972 h– (ATCC 26189), was streaked onto rich, solid YPD medium (1% yeast extract, 2% peptone, 2% dextrose, 2% agar) and incubated at 30°. From the streaked ancestor, 96 random isolated colonies were selected after 48 hr and used to found 96 MA lines. Lines were cultured six to a YPD plate, and bottlenecked by randomly selecting one isolated colony per line every 48 hr and transferring to a new plate. Lines were passaged for a total of 100 transfers (200 days). Every 10 transfers, a random colony from each line was frozen and stored in 15% glycerol at –80°.

Every 10 transfers, photographs were taken of all 96 MA lines. From these photographs, colony size was measured for five random colonies per line using ImageJ (Schneider *et al.* 2012). In addition, the numbers of cells per colony for colonies of various sizes were recorded at transfer 10 (T10) and 100 (T100) by suspending a single colony in 1 ml of water, and counting individual cells using a hemocytometer. These measurements were used to determine standard curves of colony size *vs.* cell number at these two transfers. Measurements of average colony sizes at every 10th transfer were then used to calculate the average number of cells at those transfers. From the average number of cells at every 10th transfer, the number of cell generations per transfer was estimated, using the fact that, with no mortality, the number of cells in a colony is equal to 2*^g^*, where *g* is the number of generations. The number of generations per transfer was then multiplied by the number of transfers to estimate the number of generations across the entire experiment. The effective population size across the experiment was determined by calculating the harmonic mean of the number of cells present each generation, assuming cell number doubles each generation between transfers, at which point it bottlenecks to one cell.

### Sequencing

MA lines were cultured from frozen T100 stock on solid YPD medium at 30° for 48 hr. A single colony from each line was selected, inoculated into 3 ml liquid YPD, and incubated on a rotator at 30° for 48 hr. Cells were then pelleted and DNA was extracted using the YeaSTAR kit (Zymo Research) protocol with chloroform, and an extended digestion time with zymolase of 2.5 hr at 37°. Whole genome shotgun libraries were prepared by the Georgia Genomics Facility using the Kapa Library Low-Throughput Library Preparation Kit with Standard PCR Amp Module KK8232 with dual SPRI size selection cleanup to generate 100 bp paired-end fragments with ∼300 bp inserts. After seven cycles of PCR, the libraries (96 haploid MA lines and two haploid ancestor samples) were pooled across two lanes of Illumina HiSequation 2000 machines (raw sequences are deposited at NCBI, BioProject SRP065886).

### Quality control (QC), mapping, and identification of mutations

Sequence reads from each library were quality controlled with the ea-utils and fastx toolkit in order to remove low quality reads and residual adaptor sequence (Gordon and Hannon 2010; Aronesty 2011)(Workflow deposited at https://github.com/behrimg/Scripts/blob/master/Hall_Projects/Pombe/MA_Pipeline.txt). Based on the workflow outlined in Zhu *et al.* (2014) and adjusted for a haploid dataset, quality controlled (QCed) reads were then mapped to the *Sc. pombe* reference genome ASM294v2.24 with BWA v1.1.2, sorted and indexed with SAMtools v1.0, and assigned line identification numbers with Picard Tools v1.87 (Wood *et al.* 2002; Li and Durbin 2009). Duplicated reads were marked with Picard Tools and removed, and then the remaining sequence reads were locally realigned with GATK v3.2.2 (McKenna *et al.* 2010). SNM and indel variants for each line and the ancestor were identified simultaneously using GATK’s Unified Genotyper tool with parameter settings for haploid organisms. The resulting VCF files were converted to tab delimited text using VCFtools v0.1.12a vcf-to-tab function (Danecek *et al.* 2011). All sequence differences between the MA ancestor, which was sequenced twice, and the reference were identified to determine the sequence of the ancestor. The differences between each MA line and the reference were determined, and those that were present in the ancestor were ignored. In order to call a variant, a minimum of four reads with ≥75% of the reads favoring the variant allele was needed. Regions of the genome that corresponded to centromeres, telomeres, and mating type loci (approximately 472 kbp) were excluded from the analysis to avoid inaccurate mapping. This was in addition to the two tandem rDNA repeat arrays on chromosome III accounting for 1,465 kbp, which are excluded from the reference genome. Identified SNMs and small indels were annotated using Ensembl’s variant effect predictor (VEP) while flanking regions were determined using the fill-fs program from the VCFtools package (Li and Durbin 2009; McLaren *et al.* 2010).

Presence of medium and large structural variants were investigated using the Delly software package (Rausch *et al.* 2012), and variants that passed Delly’s QC were investigated further using the integrated genome viewer (IGV) v2.1.23 (Robinson *et al.* 2011). When IGV supported a structural variant call, the variant was tested with PCR.

Sequencing also allowed the detection of across-line and other microbial contamination. Across-line contamination was deemed to have occurred if any two lines shared an identical new mutation. When this happened, one of the lines (chosen by coin flip) was discarded from the remainder of the analysis.

### Gene expression

To estimate mRNA concentrations, as a surrogate for gene expression levels for our ancestor strain, we sequenced mRNA from 10 biological replicates. We selected 10 colonies, inoculated each into 3 ml liquid YPD medium, and incubated on a rotator at 30° for 48 hr. After 48 hr, mRNA was extracted using the MasterPure Yeast RNA Purification kit (Epicentre). mRNA libraries were constructed using the Illumina Truseq mRNA Stranded Kit, amplified using 13 cycles of PCR and sequenced on an Illumina HiSequation 2500. Libraries were sequenced as 100 bp single-end reads (NCBI SRA BioProject: SRP065886). Sequenced reads were QCed in the same manner as genomic sequencing reads, reference-mapped with TopHat v.2.0.13 (Trapnell *et al.* 2009), and assembled with Cufflinks (Trapnell *et al.* 2010). The log-median fragments per kilobase of exon per million fragments mapped (FPKM) for each site across ancestor replicates was chosen to represent the level of expression at that site.

### Verification of identified mutations to determine false positive rate

Five lines were randomly selected to verify the mutations that were identified bioinformatically with Sanger sequencing (Supporting Information, Table S1). Primers were designed using Primer3 (Rozen and Skaletsky 1999) and PCR products destined for sequencing were cleaned using a standard Exo-SAP protocol (Bell 2008), and sequenced with an ABI BigDye Terminator Cycle Sequencing Kit (Applied Biosystems, Foster City, CA). Completed sequencing reactions were submitted to the Georgia Genomics Facility, and analyzed using an Applied Biosystems 3730xl 96-capillary DNA Analyzer.

### Identification of ancestor mutations across lines to determine false negative rate

Mutations present in the ancestor relative to the reference should be present in every MA line. This implies that an ancestral mutation that is not found bioinformatically in an MA line is a false negative. To estimate the false negative error rate, the total number of ancestral mutations that should have been scored (equal to the product of the number of mutations in the ancestor and the number of MA lines) was compared to the number actually scored, and the probability of missing a mutation was calculated.

### Relative mutation rate analysis

To investigate the effects of G/C content, replication time, and transcription rate, relative mutation rates were calculated across groups differing in these variables. G/C content was determined in nonoverlapping 10 kb windows across the entire genome. The number of SNMs was determined for each window and separated into 14 bins representing G/C content rounded down to the nearest 1%. Relative mutation rates were determined for each bin by determining the per base pair mutation rate for each bin and dividing by the observed genome-wide per base pair mutation rate. Bins were then separated into groups so that each group contained roughly equal numbers of SNMs, but not necessarily equal numbers of bins. Weighted averages and standard errors for each group were determined by weighting each bin by the amount of the genome covered by the bin. The same bins were used to determine relative mutation rates at A/T and G/C bases by calculating the per base pair mutation rate for just A/T and G/C bases.

Replication time was determined genome wide by assigning each SNM a replication time based on the closest origin of replication (Heichinger *et al.* 2006). SNMs were separated into 17 bins, each representing 1 min of S phase of the cell cycle. Relative mutation rates were then determined in an analogous manner as for G/C content.

Transcript abundance was determined genome wide and transcripts were separated into 15 bins based on abundance increasing from 0 to 3.5 in intervals of 0.25 log_10_FPKM. SNMs within genes were assigned a bin based on the transcript in which they reside; SNMs not mapping to a transcript were assigned a gene expression of 0. Relative mutation rate was then determined in an analogous manner as G/C content and replication time.

### Data availability

Scripts written to process and analyze the data are available on GitHub (https://github.com/behrimg/Scripts/blob/master/Hall_Projects/Pombe/MA_Pipeline.txt) and raw sequences are available on the Sequence Read Archive at NCBI (BioProject SRP065886). All other data is available upon request.

## Results

### MA lines

The relationship between colony area and the number of cells in the colony did not differ between transfers 10 and 100 (data not shown). The regression between colony size and transfer number indicated that average colony size declined over the course of the experiment such that the number of cells at T100 was approximately half the number at T10, indicating an average reduction in per-generation growth rate of approximately 5%. A reduction in growth rate is expected as haploid MA lines accumulate deleterious mutations. In diploid *S. cerevisiae*, a trend toward reduced colony size was not observed (Joseph and Hall 2004), presumably because new mutations accumulated in the heterozygous state and thus had a smaller effect on fitness due to masking. The average number of cells in a colony was used to estimate the average effective population size in a MA line as 10.26 cells. This small effective population size implies that deleterious (beneficial) mutations with a fitness effect greater than ∼0.097 would be underrepresented (overrepresented) due to the action of selection (Hall *et al.* 2008; Lynch *et al.* 2008). Mutations with smaller fitness effects are expected to accumulate approximately at random in the MA lines. The average number of cells in a colony was also used to estimate that the MA experiment lasted 1952 cell generations.

Mapping of sequencing reads to the *Sc. pombe* reference genome revealed that coverage was uniform across all three chromosomes except for telomeric regions in those lines with average sequencing depth below 43x, where telomeric sequences appeared to be affected by amplification bias (Figure S1). After sequencing and identification of nucleotide variants, we discarded 17 of the 96 MA lines due either to contamination by a different microbial species (six lines) or to across MA line contamination (11 lines), which left 79 lines for further analysis. We note that for most of the contaminated lines (10 of 11), all nucleotide variants were shared, indicating that contamination likely occurred either at one of the last transfers, or when the lines were removed from the freezer for DNA sequencing.

Approximately 472 kbp, which is ∼3.8% of the sequenced genome, was excluded from the analysis to ensure precise identification of new mutations. This included the low-complexity sequence, comprising centromeres, telomeres, the mating type region, and the representative rDNA repeat belonging to the two tandem rDNA repeat arrays on chromosome III, which had been previously excluded from the 12.47 Mb reference genome (Wood *et al.* 2011). The exact coordinates of the regions removed from the analysis are listed in Table S2.

### Differences between the MA ancestor and *Sc. pombe* reference genome

**Figure 1:**
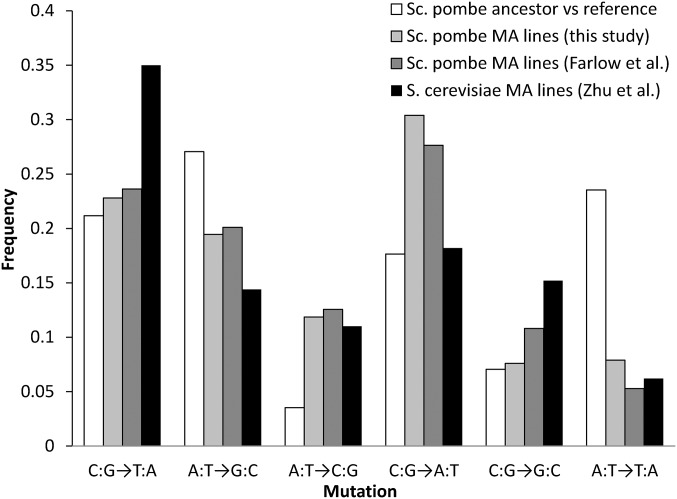
Summary of mutations for each of six possible nucleotide changes for the ancestor (ATCC 26189) *vs.* the reference, the 79 mutation accumulation (MA) lines in this study, and the 96 Farlow *et al.* (2015) MA lines. Also shown are the mutation frequencies observed in the 145 diploid *Saccharomyces cerevisiae* MA lines (Zhu *et al.* 2014).

Sequencing of the ancestor revealed a total of 262 differences between it and the reference genome. These differences included 80 SNMs, 120 small insertions (< 50 bp), 42 small deletions (< 50 bp), six double SNMs, and 14 complex mutations (Figure S2). We define double SNMs as two SNMs that occur within 50 nucleotides of one another. Complex mutations are three or more SNMs and/or indels occurring within 50 nucleotides of one another. Both complex mutations and double SNMs were deemed to be nonindependent events and were thus analyzed separately (Schrider *et al.* 2011). Of the 80 SNMs, 39 were transitions and 41 were transversions, giving a transition to transversion ratio of 0.95. Within transitions, after correcting for genomic G/C content, and assuming that all changes were mutations from the reference strain sequence to the ancestral strain sequence, G:C → A:T or T:A mutations were 1.38 times more frequent than A:T → G:C or C:G mutations (Figure 1).

The distribution of SNMs across chromosomes was not significantly different from the expectation based on chromosome length (X^2^ = 5.45, *P* = 0.06). This result held whether we tested all three chromosomes, or just chromosomes I and II (X^2^ = 3.05, *P* = 0.06), which together represent 81% of the genome. The distribution of small insertions was nonrandom, with chromosome I (5.58 Mb) containing 68, which is greater than the 53 expected based on chromosome length (X^2^ = 7.603, *P* = 0.006). Other mutations, including small deletions, complex mutations, and double SNMs, did not show bias across chromosomes, after accounting for length (Figure S3).

### Differences between the MA lines and the MA ancestor

#### Single nucleotide mutations:

**Figure 2:**
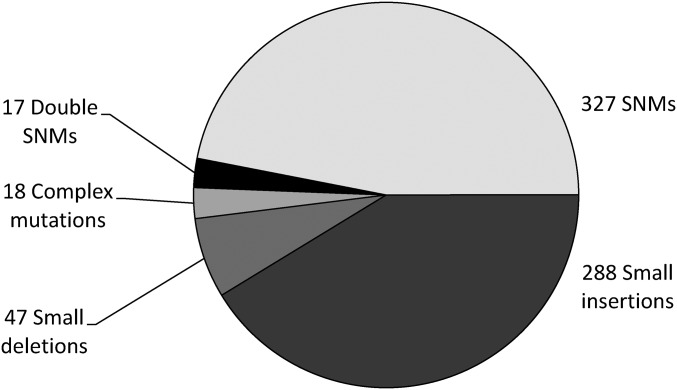
Summary of types of mutations identified across 79 MA lines. SNM, Single nucleotide mutation.

Across the 79 MA lines, 326 SNMs arose during MA, which gives the SNM rate (± 1 SE) for *Sc. pombe* as 1.70 ± 0.13 × 10^−10^ per base per generation (Figure 2 and Table S3). The SE of the estimate is based on the variance across MA lines. This mutation rate estimate does not include the 123 SNMs that were in double or complex mutations. Including SNMs in double and complex mutations increases the genome-wide mutation rate estimate to 2.34 ± 0.23 × 10^−10^ per base per generation.

**Figure 3:**
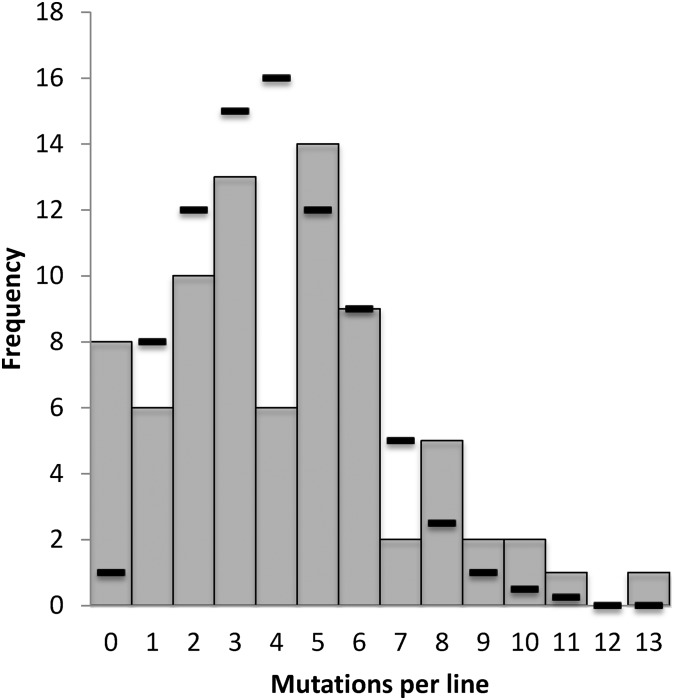
Histogram of SNM numbers per line across the 79 MA lines. The distribution is consistent with a negative binomial (*P* = 0.95, χ^2^ test). Dashes represent the expected numbers for the best-fit negative binomial distribution (mean = 4.13, overdispersion = 2.06). The total number of SNMs across all 79 MA lines is 326.

The number of SNMs varied across the 79 MA lines from zero (eight lines) to 13 (one line), with an average of 4.13 SNMs per line (ignoring doubles and complex mutations). Surprisingly, the distribution of mutations across the MA lines was not consistent with a Poisson distribution (χ^2^; *P* < 0.001). A negative binomial distribution (Gamma-Poisson), with mean = 4.13 and overdispersion parameter = 2.06, could not be rejected (χ^2^; *P* = 0.954, Figure 3). Using Akaike’s information criterion (AIC), the negative binomial was a substantially better fit to our data than the Poisson (Poisson AIC = 15.58, negative binomial AIC = 4.45; Poisson is 0.0034 as likely to explain the data). SNMs occurred at random with respect to protein-encoding genes: 52.4% of SNMs were in the 57% of the genome that is protein coding (Fisher’s exact, *P* = 0.27), and 3.6% of SNMs were in the 3% of the genome that is intronic sequence (Fisher’s exact, *P* = 0.83), suggesting that selection was inefficient during MA.

The distribution of SNMs and all other mutation classes (small insertions, deletions, complex, double SNMs) across the three chromosomes were not significantly different from the expectation based on chromosome length.

#### Single nucleotide mutation biases:

Ignoring SNMs in complex and double mutations, we observed a transition to transversion (Ts/Tv) bias of 0.72 (Figure 1). Within transitions, G:C → A:T mutations were 2.02 times more frequent than A:T → G:C. Within transversions, G:C → T:A mutations were 4.55 times more frequent than A:T → C:G. Across transitions and transversions, G/C → A/T mutations were 2.97 times more frequent than A/T → G/C mutations. If the mutational process were the sole determinant of the G/C content in *Sc. pombe*, the equilibrium genome G/C content would be 25.14%, far less than the 36.06% observed in the reference genome.

G/C content has been shown to affect local mutation rate in rodents (Hurst and Williams 2000). However, in this study, we found no effect of G/C content on the local mutation rate. We determined the G/C content in 10-kb windows across the genome, and then separated the data into groups such that each contained a similar number of SNMs. Regardless of whether we split the data into two or three groups, we found no significant difference in mutation rate across them, suggesting that local G/C content has no effect on local mutation rate. Data for the two-group case where the division is for a GC content of 36% is shown in Figure S4A.

In humans, late replicating regions have higher mutation rates (Stamatoyannopoulos *et al.* 2009). We tested for a similar relationship and found that replication time did not affect mutation rate. We assigned each nucleotide in the genome a replication time based on the time at which the closest origin of replication (ori) initiates replication during mitosis (Heichinger *et al.* 2006). There is little variation in the replication initiation times of different oris in *Sc. pombe*, with all oris firing between 68 and 85 min after release from G2 arrest (Heichinger *et al.* 2006). Further, published initiation times are reported in 1-min intervals, and so we could not split times into smaller intervals. We separated replication times into three groups and found no significant differences in mutation rate for different replication times (Figure S4B).

Mutation rate has been shown to correlate with gene expression in *S. cerevisiae* and humans (Park *et al.* 2012), but see Zhu *et al.* (2014). We found that mRNA levels were not associated with mutation rate. Using RNA-seq data collected from our ancestor line, we examined whether gene expression, measured as transcript abundance, influenced local mutation rate. Transcription rates were split into 15 bins that were then separated into two groups. These two groups did not differ in mutation rate. Adding rRNA and tRNA genes, and assuming they are expressed in the highest bin, did not alter this conclusion (Figure S5).

**Figure 4:**
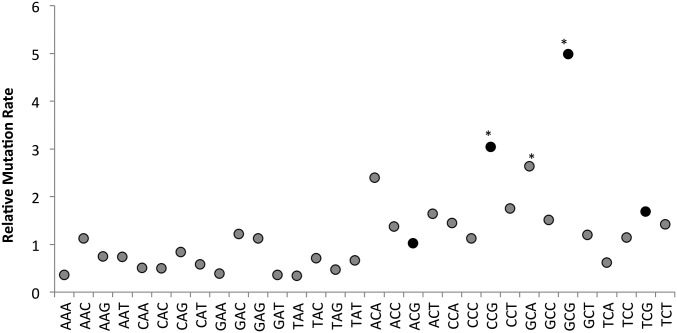
Mutation rate is affected by context. Relative SNM rate adjusted by its trinucleotide context. Trinucleotide classes represent mutation rates of both strand orientations. For example, aCa trinucleotide class includes overall mutation rate at aCa and tGt sites. Mutation rate is shown relative to the average SNM rate across all sites (= 1.7 × 10^−10^ per base, per generation). The average, relative mutation rate of 1.8 at G:C bases shows clear overall elevation over the corresponding rate of 0.66 at A:T bases. The four black-filled points are those trinucleotides with a C in the central position with a G as the 3′ neighbor. * *P* ≤ 0.003 Bonferroni corrected *t*-test.

We did find an effect of trinucleotide context, which is the identity of the nucleotides immediately before and after the mutated nucleotide, on mutation rate (Zhu *et al.* 2014). Each nucleotide position within the genome, along with its neighboring bases, was assigned to one of the 64 trinucleotide possibilities. Ignoring strand orientation allowed us to reduce the 64 possibilities to 32 groups, defined such that complementary trinucleotides belong to the same group (*e.g.*, GCA and TGC are in the same group). Mutation rates at the center position across the 32 groups were quite variable (Figure 4). We analyzed the 16 groups with A (or T) at the center position separately from those with G (or C) because C:G base pairs have a higher mutation rate than A:T base pairs, as is clearly apparent in Figure 4. After correcting for multiple comparisons, we found context did not affect mutation rate at A:T base pairs. In contrast, at C:G base pairs, mutation rates were increased in the CCG (equivalent to CGG), GCG (equivalent to CGC), and GCA (equivalent to TGC) trinucleotide groups (*t*-test; *P* = 0.0003, *P* = 0.0001, and *P* = 0.0009 respectively; Bonferroni corrected critical *P*-value = 0.0031). Interestingly, two of these trinucleotides are two of the four trinucleotide groups that contain a CpG dinucleotide, where the C is the at the center position. If increased CpG mutation rate was due to deamination of methylated cytosine (giving thymine), then we would expect CpGs to mutate to TpGs. However, of the 44 mutations occurring at CpG sites, 23 were C → A mutations (52.3%), 18 were C → T (40.1%) and three were C → G (6.6%); which is no different than expected based on the mutational spectrum of *Sc. pombe* (X^2^ = 1.76, *P* = 0.41). For the GCA trinucleotide group, C → A mutations were overrepresented relative to the mutational spectrum (18 of 22 mutations were C → A, 11 were expected; X^2^ = 8.91, *P* = 0.011).

#### Small insertion and deletion mutations:

Across the MA lines, we identified 335 small indels of less than 50 bp, including 288 insertions and 47 deletions. A complete list of indels is in Table S4. Average insertion size was 1.6 bp, while average deletion size was 3.1 bp, with insertions occurring six times more frequently than deletions, resulting in a net gain in DNA sequence across all lines of 340 bp. Small indels occurred as frequently as SNMs across the MA lines, and the resulting spontaneous indel rate, 0.174 × 10^−9^ indels/base/generation, is essentially identical to the mutation rate calculated for SNMs, ignoring double SNMs and complex mutations. Indels, however, were not randomly distributed with respect to genomic features. They were substantially underrepresented in protein coding sequence (observed: 33, expected: 191; Fisher’s exact test: *P* < 0.001), occurred as frequently as expected in introns (observed 21, expected 10; *P* = 0.064), and were over represented in noncoding regions (observed 288, expected 134; *P* < 0.001).

#### Effects of SNMs and indels:

**Figure 5:**
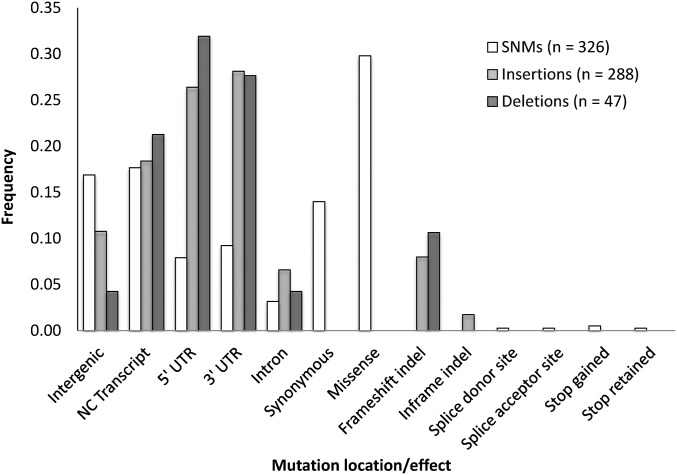
Summary of the locations and predicted functional consequences of the 326 SNMs (excluding SNMs in double and complex mutations), 288 small insertions and 47 small deletions accumulated in the MA lines. To facilitate comparison across the three mutational classes, frequencies are shown. NC, noncoding; UTR transcribed, untranslated region.

We annotated the expected functional effects of our SNM and small indel mutations using Ensembl’s VEP software (McLaren *et al.* 2010). For the 183 SNMs that occurred within coding regions, 53 were synonymous, 113 were missense, 12 were within introns, one was within a splice donor site, one was within a splice acceptor site, two were nonsense, and one changed a termination codon (UAA) into another termination codon (UAG) (Figure 5). The ratio of synonymous to missense changes (0.398) was higher but not statistically different from the expected ratio (0.323), computed using randomly generated protein coding sequences (Z-test for proportions: 1.104, *P* = 0.271) (Figure S6) (Graur 2003). This suggests selection did not substantially reduce the number of missense mutations captured during MA.

Among the 54 small indels that occurred within protein and intronic coding sequence, seven were deletions, of which five were frameshift deletions and two were within introns. For the remaining 47 insertions, 23 were frameshift insertions, five were in-frame insertions ranging from 6 to 12 bp in length, and 19 were within introns (Figure 5). The proportion of indels in protein coding sequence that were in-frame was not significantly different from 1/3 (Fisher’s exact test *P* > 0.2). Again, these results suggest that selection did not substantially reduce the number of frameshift mutations captured during MA.

#### Double mutations, complex mutations, and structural variants:

As expected, no aneuploids were detected among the haploid MA lines. We specifically searched for other structural variants including medium-sized deletions (50–1000 bp), inversions, translocations and duplications using the DELLY software that predicts structural variants from short insert paired-end sequences (Rausch *et al.* 2012). We detected three medium-sized deletions, two of which were flanked by a number of SNMs (Table S5). In addition to multiple SNMs in the region of the three medium deletions, 16 other complex mutations and 17 separate double SNMs were also detected (Table S6 and Table S7). Complex mutations thus occurred at a rate of 8.32 × 10^−12^ per base per generation and double SNMs occurred at a rate of 8.84 × 10^−12^ per base per generation.

### Error in identification of mutations

Our *Sc. pombe* lines were haploids and our sequencing coverage exceeded 25x in every line (average = 44x). Given previous work in diploid *S. cerevisiae*, we predicted we would have a very low likelihood of incorrectly calling a SNM or indel. To confirm a low false positive error rate, we randomly chose five lines and checked all predicted mutations using Sanger sequencing. Together, these lines contained 15 SNMs, 20 small insertions, three small deletions, one double SNM and one complex mutation. Sanger sequencing confirmed all of the mutations identified from next-generation sequencing, suggesting that the false positive error rate is likely is no larger than 0.025, and could be close to zero. We did find that, in the region of the complex mutation (MA Line 58, Chromosome II, positions 4,273,519–4,274,319), additional mutations not identified in next-generation sequencing data were present (*i.e.*, false negatives). These mutations were probably not detected due to the difficulty in reference mapping sequencing reads that are too divergent from the reference genome. This conjecture is supported by the extreme loss of coverage that was observed in this 800-bp region. Just 80 bp on either side of the region coverage was 48x, while across the complex mutation coverage averaged 9x, with three nucleotides in the center only being represented by a single read. Our results for this complex mutation suggest that the numbers of detected changes present in the five complex mutations we identified between the ancestor and reference strain and the 18 in the MA lines, are underestimates of the number actually present.

We also examined the probability of false negatives for SNMs. This was done by asking how many of the 272 mutations that differ between the reference and the ancestor were not found in each of the 79 MA lines. Any mutation present in the ancestor that is not identified in an MA line is a false negative, assuming the probability that a mutation converting the ancestor base back to the reference base at any of these sites is negligible. Given our estimate of the mutation rate, the probability of a mutation occurring at any of these sites in any line during the experiment is ∼0.007, indicating that failure to detect a site that differed in the ancestor is almost certainly a false negative. For SNMs, the false negative rate was 0.0001, while for small insertions and small deletions it was 0.011 and 0.017, respectively. Thus, we may have underestimated the base substitution, small insertion and small deletion mutation rates by 0.01, 1.1 and 1.7%, respectively. No mutations were found in three or more MA lines that were not also found in the ancestor, indicating that we likely detected all of the mutations in the ancestor.

## Discussion

### Rate of mutation at single nucleotides

We expected to observe a higher SNM rate in *Sc. pombe* than in *S. cerevisiae*, based on data using reporter genes (Lynch 2010). Our estimate of the base pair mutation rate in *Sc. pombe*, from the 326 accumulated SNMs, is 1.70 ± 0.13 × 10^−10^ per base per generation, which is almost identical to the mutation rate of 1.67 × 10^−10^ per base per generation, based on 867 accumulated SNM mutations, in *S. cerevisiae* (Zhu *et al.* 2014). Our estimate is slightly, but significantly, lower than the 2.00 ± 0.10 × 10^−10^ mutation rate per base per generation estimate reported by Farlow *et al.* (2015), but that estimate did not remove SNMs in double and complex mutations, except for in flocculation-related genes. Including the 123 SNMS that we observed in double and complex mutations raises the genome-wide mutation rate estimate to 2.34 ± 0.23 × 10^−10^ per base per generation, which is not significantly different from the Farlow *et al.* (2015) estimate. In both studies, there was no effect of either local G/C content, transcript abundance, used as a proxy for gene expression, or timing of replication on the local mutation rate.

With our estimates of the mutation rate and the genome-wide estimate of π from Jeffares *et al*. (2015), we can calculate the effective population size for *Sc. pombe* as either 6.52 × 10^6^ (including SNMs in double and complex mutations) or 8.80 × 10^6^ (ignoring SNMs in double and complex mutations), which span the estimate of effective population size for *S. cerevisiae*, 8.53 × 10^6^ (Liti *et al.* 2009). Thus, *Sc. pombe* and *S. cerevisiae* do not seem to differ very much in their effective population size, suggesting that selection will encounter the drift barrier (Sung *et al.* 2012a), the point at which the fitness advantage of new mutation is the same magnitude as the strength of genetic drift, at similar selection coefficients in both species. We note that our population size estimates are smaller than the estimate of 12 million reported in Farlow *et al.* (2015), the reason being that they utilize π calculated from intergenic regions, which is larger (0.0048) than the genome-wide π (0.0030) that we use here.

### Bias in single nucleotide mutations

There are both similarities and differences between the SNM mutation biases in *Sc. pombe* and *S. cerevisiae*. The most obvious difference is that the most common SNM in *S. cerevisiae* is a C:G → T:A transition, while in *Sc. pombe* it is a C:G → A:T transversion (Figure 1). In addition, C:G → G:C transversions are more common, and A:T → G:C transitions less common in *S. cerevisiae* than in *Sc. pombe*. In contrast, both A:T → C:G and A:T → T:A transversions are similarly frequent in *S. cerevisiae* and *Sc. pombe* (Figure 1). Both species show an elevated rate of G/C → A/T, which points to oxidative damage as a major cause of mutagenesis (Cheng *et al.* 1992; Shen *et al.* 1994). Within our MA lines, G/C → A/T mutations occurred at a rate that was ∼three times greater than A/T → G/C mutations, which is the same as the bias observed by Farlow *et al.* (2015), and higher than the ∼two-fold bias seen in *S. cerevisiae* (Zhu *et al.* 2014). The equilibrium G/C content calculated from the mutation spectra is 25% and 32% in *Sc. pombe* and *S. cerevisiae*, respectively.

In both species the observed G/C content (35% in *Sc. pombe* and 38% in *S. cerevisiae*), is higher than predicted from mutation biases, suggesting that there is either selection for lower G/C content or some other mechanism, perhaps biased gene conversion, or selective constraint on protein sequence, which is causing the increase in genomic G/C content. Biased gene conversion is unlikely given the rarity of heterozygote formation in *Sc. pombe* (Farlow *et al.* 2015). Suggestive evidence for an additional force is present in the ancestor, whose G/C → A/T bias relative to the reference strain is 24% less than observed in the MA lines, indicating that G/C → A/T mutations are fixing at a lower rate than expected.

### Rate and spectrum of indels

In our MA lines, we observed a 6.13-fold insertion bias. Insertions accounted for 86% of small indel events, consistent with previous observations of the GT repeat region in the *ade6* gene of *Sc. pombe*, where 83% of indel events in wild-type were insertions (Mansour *et al.* 2001). This bias is identical to that observed by Farlow *et al.* (2015). A similar bias toward insertions has also been observed in *C. elegans* (3.75 insertions to deletions) (Denver *et al.* 2004) and haploid, but not diploid, *S. cerevisiae* (3.55 insertions to deletions) (Lynch *et al.* 2008). We do not know of any mechanism that would predict more insertion mutations based on ploidy, but if small deletions are more often strongly deleterious than small insertions, they would be underrepresented in MA experiments in haploid species, and in diploid species passaged with strong inbreeding (*e.g.*, *C. elegans*). In haploid *S. cerevisiae*, despite their rarity, the size of small deletions was able to negate the effect of small insertions in terms of changes in genome size. This is not the case in *Sc. pombe*. Even though small deletions were twice as large as small insertions, small insertions occurred six times more frequently resulting in a net gain of 340 bp across the genome across all MA lines. This suggests that *Sc. pombe*’s current genome size is not at equilibrium with respect to an insertion/deletion mutational balance. Additionally, in the ancestor, small deletions may have been favored by selection: 17.3% of the differences between the ancestor and reference are small deletions compared to only 6.7% in the MA lines, which is a significant difference.

Similar to observations in *C. elegans*, *Sc. pombe* experiences small indels as often as SNMs (Denver *et al.* 2004), resulting in an indel rate that is 10-fold higher than haploid *S. cerevisiae* and almost 35-fold higher than diploid *S. cerevisiae* (*Sc. pombe*: 1.74 × 10^−10^ *vs.* haploid *S. cerevisiae*: 0.2 × 10^−10^, and diploid *S. cerevisiae*: 0.05 × 10^−10^ per base pair per generation). One hypothesis for the differences in indel rate may be due to differences in genome complexity between the two species. *Sc. pombe*’s genome is 60.2% protein coding (57% excluding introns) while *S. cerevisiae*’s is 71% protein coding (70.5% excluding introns) indicating a higher prevalence of noncoding DNA in *Sc. pombe*. Most indels identified in *Sc. pombe* and *S. cerevisiae* are within low complexity, intergenic regions such as microsatellites and mononucleotide runs. However, in an analysis of short simple repetitive sequences, there is little observed difference in the amount of repetitive sequence in *S. cerevisiae* and *Sc. pombe* (Karaoglu *et al.* 2005). An alternate hypothesis is that there are differences in mismatch repair pathways between these two species. However, both species contain MSH2 homologs, which are responsible for high fidelity repair of small indels (Rudolph *et al.* 1999), suggesting that this hypothesis would require differences in fidelity of the mismatch repair pathway, rather than presence *vs.* absence. A third hypothesis is that there may be differences in polymerase fidelity between *Sc. pombe* and *S. cerevisiae* causing differing rates of strand slippage resulting in *Sc. pombe*’s increased insertion rate. However, given similar effective population sizes, we do not expect fidelity to differ in these two species. Finally, the difference may be due to detection issues, especially the problem of false negatives. Our study yielded an indel rate that is three times as large as that reported by Farlow *et al.* (2015), which might be explained by false negatives. The methods of sequence mapping and variant calling differ between the two studies. Assuming there is no substantial difference in indel mutation rate between the two strains, the Farlow *et al.* (2015) study should have accumulated approximately 354 indels. Instead, only 119 were discovered. This difference suggests that our methods reliably identify a greater percentage of the mutations (*i.e.*, we have fewer false negatives). We directly estimated our false negative error rate by determining whether an ancestral mutation, of which there were 272, was detected in all MA lines, which represents a total of 21,488 mutations tested across the 79 MA lines. For small insertions and deletions, the false negative error rates were 1.1% and 1.7%, respectively. A direct estimate of the false negative error rate was not performed in the study of Farlow *et al.* (2015), though they did introduce simulated SNMs into their reference assemblies to indirectly estimate a false negative error rate for SNMs. If the difference in indel rate estimates between the two studies is due to false negatives, then the Farlow *et al.* (2015) study missed about two thirds of all indels.

### Complex mutations, double mutations, and aneuploidy

We also predicted an increase of mutations associated with double-stranded breaks; particularly double SNMs and complex mutations. Double-stranded breaks are lethal to a cell unless repaired. Repair can involve homologous recombination, which tends to be the preferred mechanism (Raji and Hartsuiker 2006), but can also utilize nonhomologous end-joining (NHEJ). Recombinational repair requires homologous copies of DNA. Compared to *S. cerevisiae*, *Sc. pombe* has 2.5 times fewer homologous copies available on average for recombinational repair of a double-strand break, and sometimes has no copies present (see *Introduction*). In the absence of homologous DNA, double-stranded breaks are repaired through NHEJ. Homologous recombination is considered an error-free method of repair, while nonhomologous end joining is considered to be error-prone, introducing small insertions or deletions when partially degraded ends inhibit precise repair (Cavero *et al.* 2007; Shrivastav *et al.* 2008).

The locations of complex mutations were not random. Six of the 18 complex mutations occurred within three of the nine flocculin genes. All of these six mutations occurred within the characteristic tandem repeats found in these genes (Verstrepen *et al.* 2005). The flocculin tandem repeats are known to cause replication errors and are highly prone to double-stranded breaks and, as a result, recombination (Verstrepen *et al.* 2005). Their propensity for double-stranded breaks could explain the repeated observation of complex mutations within them if complex mutations are, in fact, caused by mutagenic NHEJ repair of double-stranded breaks. To make sure that inferred mutations in the flocculin genes were not caused by gene conversion/recombination, we checked for and found no evidence of recombination between paralogs. An excess of mutations in flocculin genes was also observed by Farlow *et al.* (2015). They interpreted this finding as indicating positive selection for some unknown flocculation phenotype on their plates. They noted that only one mutation mapped to flocculation-related genes in *S. cerevisiae* (Zhu *et al.* 2014), suggesting that an increased mutation rate was not the underlying cause of the observed overrepresentation of mutations in these genes. However, mapping to flocculation genes in *S. cerevisiae* is problematic (M. G. Behringer, unpublished data) due to their repetitive nature, so mutations in these genes may have been missed in that study. In addition, given our estimate of the small effective population size within MA lines (10.26 cells), and the location of mutations in the MA lines indicating ineffectual selection, we favor increased mutation rate, caused by a high propensity for double-stranded breaks, as the explanation for more mutations occurring in these genes.

As we predicted given its haploid state, possession of only three chromosomes, and previous work (Niwa *et al.* 2006), there were no instances of aneuploidy. In *Sc. pombe*, aneuploidy has only been observed as a disomic haploid of chromosome III. Even if a disomic haploid had been fixed in a line at an intermediate transfer, it would likely have been unstable (Niwa and Yanagida 1985; Niwa *et al.* 2006) and thus rapidly lost. Interestingly, when *S. cerevisiae* is passaged as a mitotic haploid it tends to be very unstable (Lynch *et al.* 2008), and at large population size, where selection is effective, it reverts to a diploid state (Mable and Otto 2001). This instability is in contrast to the relative stability of diploid strains (Nishant *et al.* 2010; Zhu *et al.* 2014). This implies that aneuploidy is generally less likely to occur in a natural haploid than in a natural diploid. However, when a natural diploid is kept as a haploid strain artificially, disomies, or other steps toward diploidy, may be tolerated and perhaps even favored. It would be interesting to see if the instability of haploidy in *S. cerevisiae*, which is a natural diploid, would also be reflected in instability of a diploid *Sc. pombe*, which is a natural haploid.

### Cytosine mutation in absence of methylation

One of the major surprises of the *S. cerevisiae* mutation spectrum is the high mutation rate observed at some C:G base pairs, particularly in CpG dinucleotides found in CCG and TCG trinucleotides. We observed a similar, unexpectedly high mutation rate at some C:G base pairs, especially CCG and GCG. In many eukaryotes a major cause of mutation at cytosine nucleotides is spontaneous deamination of methylated cytosines (5-mC) to thymine, which results in a T:G mismatch that can then be repaired, or replicated, to give a C:G → T:A substitution. Deamination of methylated cytosines was hypothesized to in part explain the elevated mutation rate of C:G base pairs in *S. cerevisiae* (Zhu *et al.* 2014), in spite of the fact that 5-mC is generally thought to be absent in this species (Antequera *et al.* 1984; Proffitt *et al.* 1984). The reason for considering the possibility that CpG mutation is caused by methylation is the low level of DNA methylation reported in *S. cerevisiae* (Tang *et al.* 2012). *Sc. pombe* is thought to lack 5-mC (Antequera *et al.* 1984), though, unlike *S. cerevisiae*, it does have a DNA methyltransferase homolog, *pmt1* (Wilkinson *et al.* 1995), so methylation might occur. However, even if cytosines are methylated at a very low rate, our data suggest that methylation cannot explain the mutational bias at these sites. Methylated cytosines cause C:G → T:A transition mutations because of spontaneous deamination, not the observed C:G → A:T transversions. Additionally, the mutation biases at the C:G base pair in a CpG dinucleotide are no different from the biases at a C:G base pair that is not in a CpG context. Together, these observations suggest a mechanism other than deamination of methylated cytosines is driving the increased relative mutation rate at CpG dinucleotides. This suggests that methylation may not be the only cause of high mutation rates at CpG dinucleotides in other species. Surprisingly, the Farlow *et al.* (2015) study reported that C:G → T:A transitions were significantly more common at CpG sites than in the non-CpG context, which is in sharp contrast to our finding, where both contexts show the same spectrum of mutations.

### Spectrum of single nucleotide mutations

If differences between the ancestor and the reference represent both neutral and selected mutations, then the mutation spectrum might differ from that observed among the MA lines. In the MA lines SNMs represent 46.9% of the observed mutations, while in the ancestor they are significantly less common. The lower proportion of SNMs among differences between the ancestor and reference suggests that many of the SNMs that arose in the lineages linking the two strains were deleterious and were thus prevented from fixing. The location of the SNMs in the genome with respect to exon *vs.* intron, and noncoding regions is similar in both the ancestor and MA lines, suggesting that many of SNMs present in the ancestor relative to the reference are likely neutral (Figure 2 and Figure S6).

### Divergence of the ancestor and reference strains

Both the ancestor and the sequenced reference strains have the same strain designation, 972 h–. However, given that they differ in sequence, they must have been separately passaged for some period of time. If all 80 of the observed SNM differences between the ancestor and the reference strains are neutral, that would suggest the two strains are between 26,800 and 36,900 generations diverged, depending on the mutation rate used to estimate the number of generations. Assuming that a strain might be passaged at most 50 times per year (∼1000 cell generations), this would indicate that the strains are almost 32 years separated (16 years of independent culturing in the lab if both were being passaged). The 972 h– strain was brought into the lab in the 1940s (Leupold 1950), so this number of generations is certainly possible. Alternatively, these mutations may have accumulated during refrigerative storage, though we know of no data to test this hypothesis. Another possibility is that the ancestor and reference strains are fewer generations diverged, with many of the sequence differences having been fixed by positive selection. Selection seems to be a less likely explanation because the distribution of effects of mutational differences between the ancestor and reference is strikingly similar to that for the MA lines, where selection is known to be minimal (Figure S6).

### Reproducibility of MA experiments

Throughout, we have compared our results to those of a recently published mutation accumulation study in *Sc. pombe* (Farlow *et al.* 2015). It is striking how the results from the two studies are so similar, especially given that we used different strains (972 h– *vs.* ED668, BG_0000H8 h+), and passaged them at different temperatures (30 *vs.* 32°) on different media (YPD *vs.* YES) at different intervals (every 2 *vs.* every 3–4 days). In our study, the specifics of the protocol were chosen in order to be identical to the conditions used in the *S. cerevisiae* MA study (Zhu *et al.* 2014) to facilitate a comparison of the results between these two species. Fortuitously, 30° is the optimal growth temperature for our *Sc. pombe* isolate (http://www.atcc.org/Products/All/26189.aspx).

Comparing the two studies, the SNM mutation rate estimates are essentially identical (when we include SNMs from double and complex mutations), the SNM spectrum (Figure 1) and the indel bias are essentially identical, and mutations in flocculin genes are overrepresented. Further, neither study found an effect of G/C content, transcription levels, scored as transcript abundance, or replication timing on local SNM rate. One discrepancy between the studies, the three-fold difference in indel rate, may be due to a high rate of missed mutations in their study and may thus not be a real difference; these indels could perhaps be discovered upon reanalysis. The long list of similar findings is reassuring given that MA experiments are time-consuming to repeat. However, there is one striking disparity between the two studies. CpG mutations in the Farlow *et al.* (2015) study were biased in favor of C:G → T:A, even though C:G → A:T mutations were more common when averaged across all C:G sites. In contrast, we found C:G → A:T mutations were more common in CpG dinucleotides, and the spectrum of mutations at CpG sites did not vary from the genome-wide mutation spectrum. This is an important difference because our study indicates that it is not methylation of cytosine that is driving the higher mutation rate at CpG sites, while the results of the Farlow *et al.* (2015) study suggest that methylation could be the cause. In summary, we find that *Sc. pombe* and *S. cerevisiae* have essentially identical base pair mutation rates, which, coupled with genome-wide estimates of within-species polymorphisms, suggests their effective population sizes are similar. We observe considerable differences in the mutation spectrum and indel rate, suggesting that there are species-specific differences in factors affecting mutation bias and indel rate between these two species. Additionally, we note that the rates of small insertions *vs.* deletions predict a growing genome, suggesting either that the genome size in *Sc. pombe* is not at equilibrium, or that rare large deletions offset increases caused by small indel bias. The sample size of captured large insertions and deletions was insufficient to test this hypothesis. We also found that CpG sites are highly mutagenic, but the mutation bias at these sites is not caused by deamination of methylated cytosine, suggesting another factor makes these sites prone to mutation. The results of this MA experiment are reassuringly similar to the results reported from a recently published MA experiment using *Sc. pombe*, suggesting that these experiments can give repeatable estimates despite differences in starting strains and growth conditions.
